# Efficient Temporal Processing of Naturalistic Sounds

**DOI:** 10.1371/journal.pone.0001655

**Published:** 2008-02-27

**Authors:** Nicholas A. Lesica, Benedikt Grothe

**Affiliations:** 1 Department of Biology II, Ludwig-Maximilians-University Munich, Martinsried, Germany; 2 Bernstein Center for Computational Neuroscience, Munich, Germany; University of MarylandUnited States of America

## Abstract

In this study, we investigate the ability of the mammalian auditory pathway to adapt its strategy for temporal processing under natural stimulus conditions. We derive temporal receptive fields from the responses of neurons in the inferior colliculus to vocalization stimuli with and without additional ambient noise. We find that the onset of ambient noise evokes a change in receptive field dynamics that corresponds to a change from bandpass to lowpass temporal filtering. We show that these changes occur within a few hundred milliseconds of the onset of the noise and are evident across a range of overall stimulus intensities. Using a simple model, we illustrate how these changes in temporal processing exploit differences in the statistical properties of vocalizations and ambient noises to increase the information in the neural response in a manner consistent with the principles of efficient coding.

## Introduction

The efficient coding hypothesis suggests that sensory systems should be optimized to process signals that are typical of those experienced in the natural environment [1]. Indeed, it has been shown that auditory neurons code sounds with naturalistic statistics more efficiently than those with artificial statistics [2]–[4] and that the response properties of auditory neurons are matched to the statistics of natural sounds [5], [6]. It has been further hypothesized that adaptive mechanisms serve to maintain this efficient coding under changing stimulus conditions by altering the response properties of the system in response to changes in the statistical properties of the relevant stimulus itself or in the context in which the stimulus is presented [7].

The most widely studied adaptive mechanisms are those that modulate temporal processing in the retina (for review, see Meister and Berry [8]). Numerous studies have characterized the functional effects of these mechanisms, demonstrating, for example, that a decrease in the mean luminance or contrast of the visual stimulus evokes a change in the dynamics of temporal receptive fields (RFs) that corresponds to a change from bandpass to lowpass tuning for temporal frequency [9], [10]. Analogous changes in temporal processing have also been reported in the auditory system, where a decrease in the mean intensity or variance of the amplitude modulations (AMs) in the auditory stimulus or the addition of a broadband noise mask can evoke a change in the dynamics of temporal RFs that corresponds to a change from bandpass to lowpass tuning for modulation frequency [11]–[14].

Most studies of adaptive temporal processing are based on responses to artificial stimuli such as gratings (or pure tones) and broadband noise. However, if sensory systems are indeed optimized to process stimuli that are typical of the natural environment, as suggested by the efficient coding hypothesis, then the functional consequences of adaptive processing must be evaluated by studying responses to natural stimuli directly. In this study, we investigate the ability of the mammalian auditory system to adapt its temporal processing strategy under natural stimulus conditions by analyzing responses to vocalization stimuli with and without additional ambient noise. Vocalizations and ambient noises typically have different statistical properties [15], [16] and we hypothesized that the auditory system could exploit these differences and modify its temporal processing strategy to maintain efficient coding. Using a combination of experimental and simulated responses, we demonstrate that the addition of ambient noise to vocalization stimuli does indeed evoke dramatic changes in the temporal processing strategy of neurons in the auditory midbrain, and that these changes serve to increase the information in the neural response.

## Results

### Temporal properties of vocalizations and ambient noises

**Figure 1:**
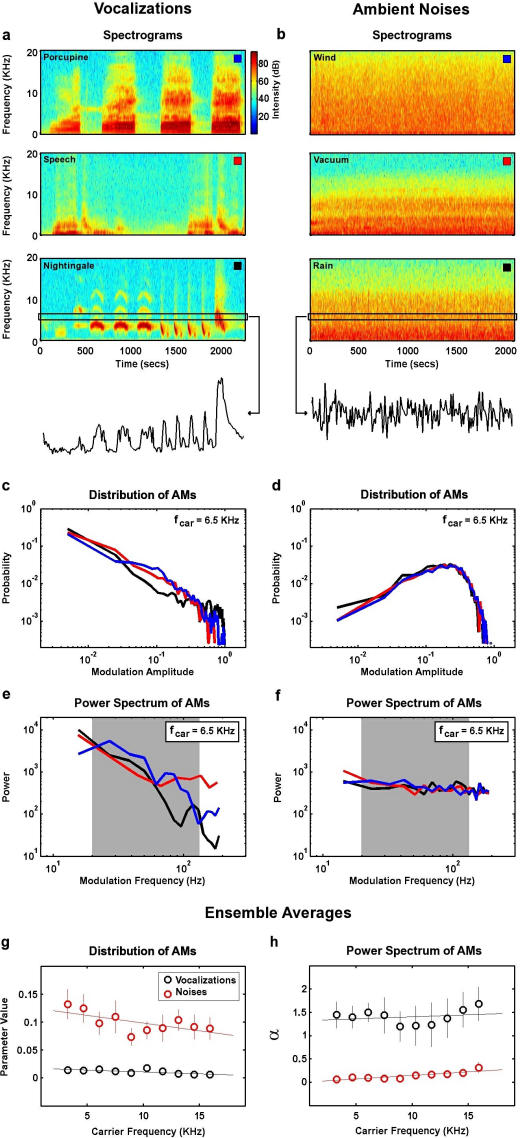
Temporal properties of vocalizations and ambient noises. a,b) Spectrograms of example sounds from the vocalization (Mexican hairy porcupine, human, and common nightingale) and ambient noise ensembles (wind, vacuum cleaner, rain), with the AMs in nightingale song and rain in the frequency band around 6.5 KHz shown below. c,d) The amplitude distributions of the AMs in the sounds shown in a and b in the carrier frequency band around 6.5 KHz. Colors correspond to the boxes in the upper righthand corner of the spectrograms in a and b. AMs were normalized to have a minimum amplitude of 0 and a maximum amplitude of 1. e,f) The power spectra of the AMs in the sounds shown in a and b in the carrier frequency band around 6.5 KHz, with the gray band denoting the 20–120 Hz frequency range. AMs were normalized as in c and d. g) The parameter values that provided the optimal fits for the average amplitude distribution of the AMs in each sound ensemble for a range of carrier frequencies (exponential distribution for vocalizations, Rayleigh distribution for ambient noises). Each sound ensemble was divided into 10 segments, and the error bars in panel g denote one standard deviation of the distribution of optimal parameter values across these segments. h) The value of *α* that provided the best fit of the function 1/*f^α^* to the average power spectra of the AMs in each sound ensemble in the 20–120 Hz frequency range for a range of carrier frequencies.

Before testing the ability of the auditory system to adapt its strategy for the temporal processing of vocalizations in the presence of ambient noise, we first investigated the statistical properties of these two classes of sounds. We compiled two ensembles of sounds containing representative examples of vocalizations (animal calls, human speech, bird songs) and sustained ambient noises (wind, vacuum cleaner, etc.). The spectrograms of several example sounds are shown in figures 1a and b.

Because we were interested in temporal processing, we focused our analysis on the AMs within narrow carrier frequency bands, as shown for the final spectrogram in each ensemble. We computed the amplitude distributions and power spectra of the AMs for a range of carrier frequency bands for each sound. The amplitude distributions and power spectra of the AMs within the frequency band around 6.5 KHz for the example sounds are shown in figures 1c–f. It is clear that the AMs of vocalizations and ambient noises have different statistical properties. As shown in figures 1c and d, the distributions of the AMs in the vocalizations decrease monotonically with increasing amplitude, while the distributions of the AMs in the ambient noises have a central peak. Furthermore, as shown in figures 1e and f, the power spectra of the AMs in the vocalizations fall off with increasing frequency (with the ‘1/f’ power law that is typical of natural sounds [15], [17], [18], indicating the presence of strong correlations, while the AMs in the ambient noises have approximately equal power at all frequencies.

To provide a characterization of the AMs that are typical of vocalizations and ambient noises, we averaged the distributions and power spectra of the AMs in each carrier frequency band across all sounds in each ensemble. To quantify the changes in the statistical properties of the AMs in each ensemble across carrier frequency, we fit the ensemble-averaged amplitude distributions and power spectra with parametric functions. The parameter values that provided the optimal fits of the amplitude distributions (exponential for vocalizations, Rayleigh for ambient noises) for a range of carrier frequency bands are shown in figure 1g. The optimal parameter values were relatively constant across the range of carrier frequencies between 2.5 and 17.5 KHz, with average values of 0.015 for vocalizations and 0.1 for ambient noises. The ensemble-averaged power spectra were fit with the function 1/*f^α^* in the 20–120 Hz frequency range (denoted by the gray band in figures 1e and f) and the values of *α* that provided the best fit of this function for a range of carrier frequency bands are shown in figure 1h. As with the optimal parameters for the amplitude distributions, the average values of *α* (related to the slope of the power spectra on logarithmic axes) were relatively constant across carrier frequencies, with average values of 1.4 for vocalizations and 0.1 for ambient noises.

### Temporal processing of vocalization stimuli depends on stimulus context

**Figure 2:**
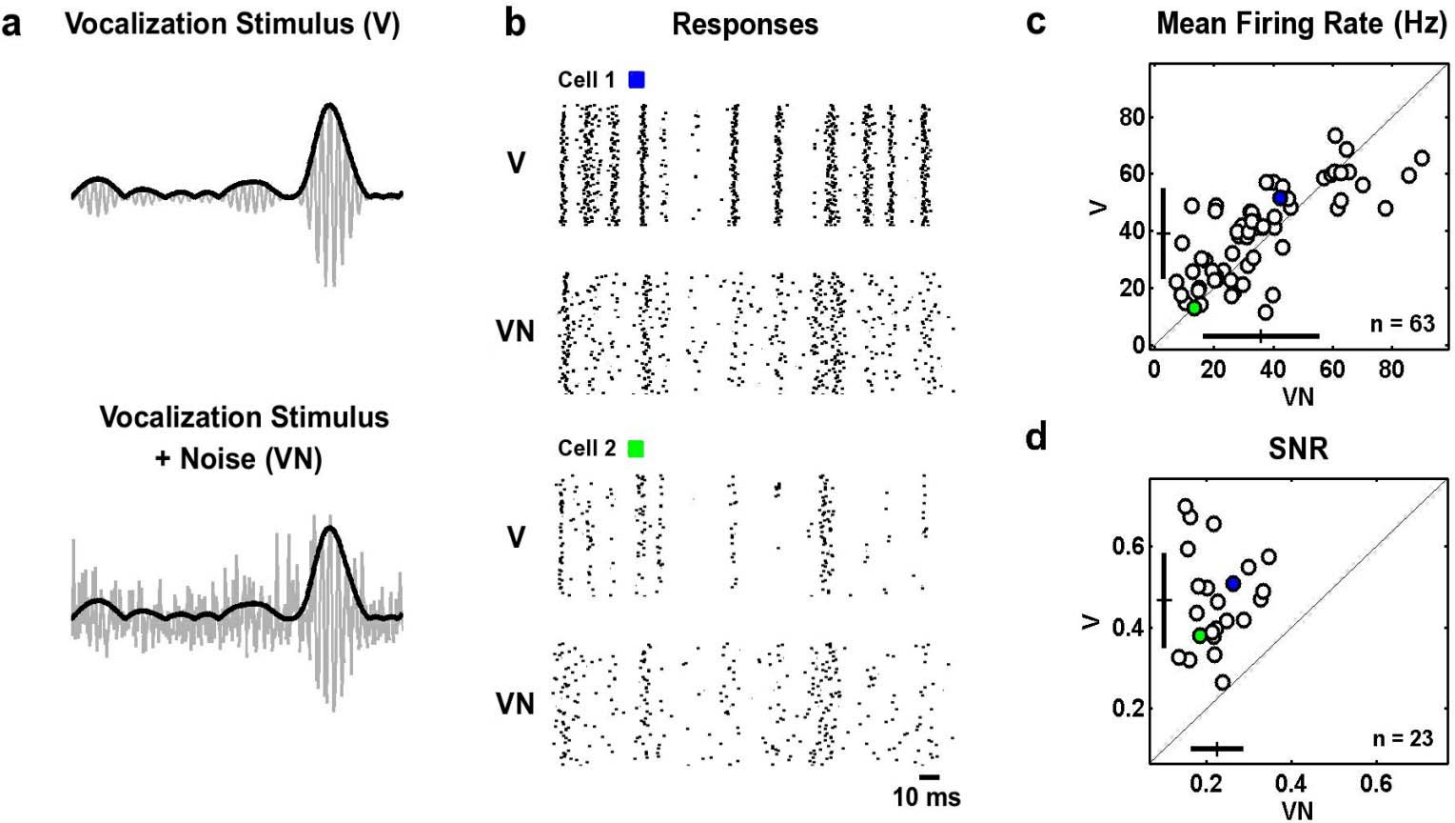
Auditory responses to vocalization stimuli with and without ambient noise. a) A schematic illustration of the two stimulus conditions used in this study: the vocalization stimulus alone (V), and the vocalization stimulus with ambient noise (VN). The V stimulus was a pure tone at the neuron's preferred frequency that was modulated by a signal with the same amplitude distribution and power spectrum as the AMs in the ensemble of vocalizations. The VN stimulus was the V stimulus added to broadband noise that was modulated by a signal with the same amplitude distribution and power spectrum as the AMs in the ensemble of ambient noises. The SNR in the VN stimulus was −10 dB. The gray lines represent the actual stimulus waveform and the black lines represent the AMs of the vocalization stimulus. b) The responses of two typical cells to repeated presentations of the V and VN stimuli. For each repetition of the VN stimulus, the vocalization signal was the same, while the ambient noise signal was different. The preferred frequencies of the cells were 2.5 KHz and 16.7 KHz, respectively. The boxes indicate the colors used to identify the mean firing rates and SNRs of these two cells in the scatter plots shown in c and d. c) The mean firing rates for a population of cells during non-repeating 40 second segments of the V and VN stimuli. The crosses denote the population mean±one standard deviation. The blue and green circles correspond to the cells for which responses are shown in b. d) The SNRs of the responses of a subset of cells to short repeated segments of the V and VN stimuli (3 seconds, 100 repetitions). SNR was calculated for firing rate responses in 1 ms bins as described in the Materials and Methods.

Based the above results, we created modulation signals with amplitude distributions and power spectra that were typical of the sounds in our vocalization and ambient noise ensembles (i.e. the vocalization signal had an exponential amplitude distribution with parameter value 0.015 and its power spectrum was 1/*f* ^1.4^, while the ambient noise signal had a Rayleigh amplitude distribution with parameter value 0.1 and its power spectrum was 1/*f* ^0.1^). We used these modulation signals to create two stimuli, denoted V and VN, with which we could characterize temporal processing of vocalization stimuli alone and in the presence of ambient noise, as shown in figure 2a.

The V stimulus consisted of a pure tone at a neuron's preferred carrier frequency with an amplitude envelope that was modulated by the vocalization signal. The VN stimulus consisted of the V stimulus added to broadband noise that was modulated by the ambient noise modulation signal. The signal to noise ratio (SNR) of the VN stimulus was −10 dB.

We made single-unit extracellular recordings of the responses to these two stimuli in the central nucleus of the inferior colliculus (IC) of anesthetized gerbils. The responses of two typical cells to repeated presentations of a short segment of the V and VN stimuli are shown in figure 2b. Across a population of cells for which we recorded responses to non-repeating 40 second segments of the V and VN stimuli, the addition of noise had no consistent effect on mean firing rate, as shown in figure 2c (V: 39.1±16.6 Hz, VN: 36.6±19.1 Hz, paired t-test, p>0.1, n = 63). However, across a subset of cells for which we recorded responses to repeated 3 second segments of the V and VN stimuli (for VN stimuli, each repeat used the same vocalization signal and a different ambient noise signal), the addition of noise caused a 49% decrease in the reliability of the response (defined as SNR for firing rate in 1 ms bins, see Materials and Methods), as shown in figure 2d (V: 0.45±0.1, VN: 0.23±0.06, paired t-test, p<0.001, n = 23).

To characterize the processing of the AMs in the V and VN stimuli, we estimated temporal receptive fields (RFs). The temporal RF is a linear filter relating the AMs in the stimulus to the neural response (i.e. convolution of the modulation signal with the temporal RF gives a prediction of the neural response). It is important to note that because the V and VN stimuli contain strong correlations (their power spectra are not flat), standard techniques for estimating temporal RFs (e.g. spike-triggered averaging or reverse correlation) can produce a biased result. To correct this bias, we used a least-squares technique which produces RF estimates that are independent of the second-order correlations in the stimulus. We also perform simulations to verify that the RF estimates were not influenced by higher order correlations of the specific stimuli used in this study (see Materials and Methods).

**Figure 3:**
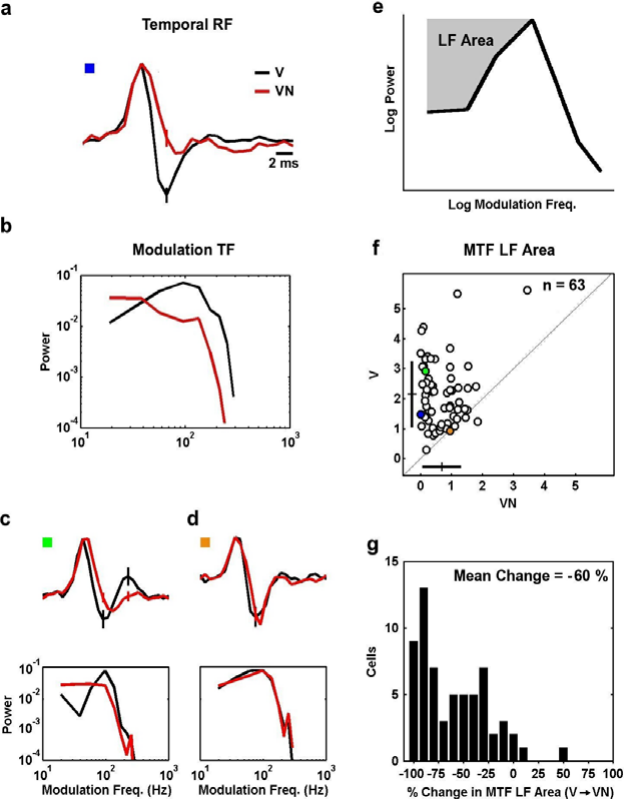
Temporal processing of vocalization stimuli with and without ambient noise. a) The temporal RFs estimated from responses to the V (black) and VN (red) stimuli for a typical cell. The preferred frequency of the cell was 12.7 KHz. The error bars represent 95% confidence bounds. The RFs were normalized to have the same peak value for plotting. b) The MTFs corresponding to the RFs in a. MTFs were obtained by computing the power spectrum of the RF. Before computing the power spectrum, RFs were normalized such that the variance of the result of the convolution of the RF with the vocalization signal was one. c,d) RFs and MTFs for two additional cells with preferred frequencies of 2.5 and 12.5 KHz, respectively. RFs and MTFs were normalized as described in a and b. e) The area above the MTF corresponding to the attenuation of low frequencies was measured on logarithmic axes, from the lowest non-zero frequency measured (20 Hz) to the peak. Before calculation of the LF area, the MTF was normalized to have a peak value of one. The LF area was zero for those neurons whose MTFs were monotonically decreasing. f) The LF area of the V and VN MTFs for the population of 63 cells. The crosses denote the population mean±one standard deviation. The colored circles correspond the RFs and MTFs shown in a–d as indicated by the colored boxes. g) A histogram showing the percent changes between the LF areas in f.

The temporal RFs of a typical cell during stimulation with the V and VN stimuli in figure 3a. The V RF (black) exhibits fast, biphasic dynamics when coding the vocalization stimulus alone, while the addition of noise evokes a change to the slower, more monophasic dynamics of the VN RF (red). As shown in figure 3b, these changes are also evident in the frequency domain representation of the temporal RF, the modulation tuning function (MTF), which reflects the response of the neuron to different modulation frequencies: the V RF has bandpass modulation tuning (black), while the tuning of the VN RF is lowpass (red). The effect of the addition of noise to the vocalization stimulus on temporal processing varied widely, with the MTFs of many neurons changing from bandpass to pure lowpass (figures 3a, b, and c), while the MTFs of other neurons did not change at all (figure 3d). We quantified these effects by measuring the area above the MTF (on logarithmic axes) that corresponds to the attenuation of low frequencies, as shown in figure 3e. Across the population, the LF areas of the VN MTFs were significantly smaller than those of the V MTFs, as shown in figure 3f (paired t-test, p<0.001, n = 63). The average decrease in MTF LF area was 60% (see histogram in figure 3g), indicating that, as a population, IC neurons exhibit a strong shift from bandpass modulation tuning under noise-free conditions to lowpass modulation tuning with the addition of noise.

**Figure 4:**
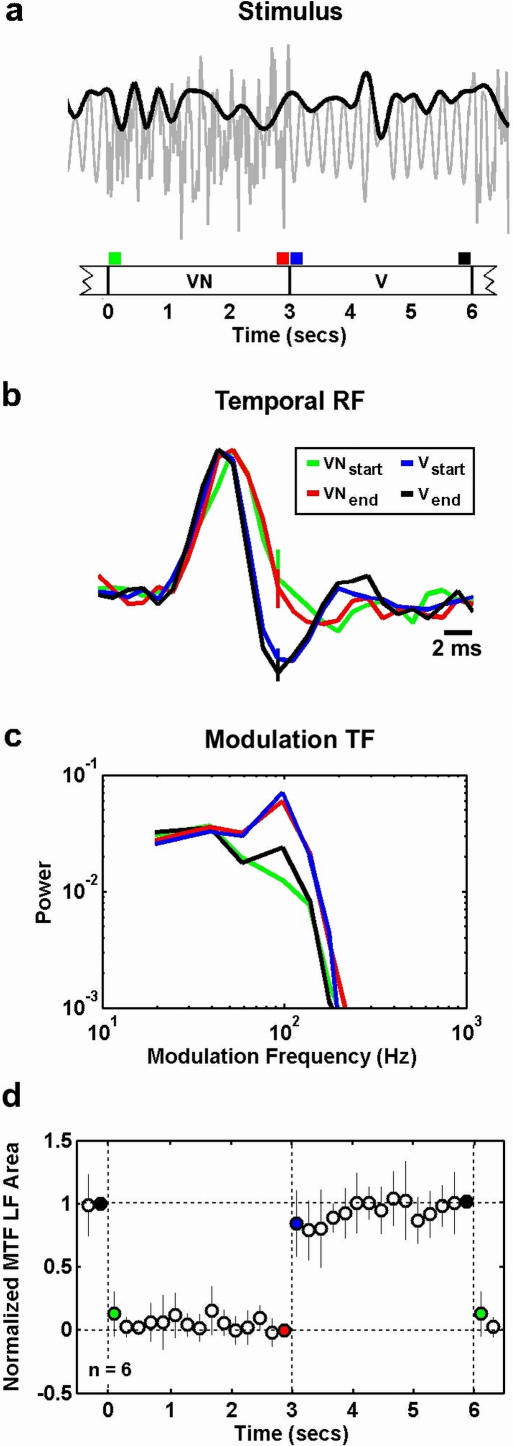
Rapid changes in temporal processing are evoked by the onset or offset of ambient noise. a) A schematic illustration of the stimulus, which switched between V and VN every 3 seconds. The gray line represents the actual stimulus and the black line represents the vocalization modulation signal. b) The temporal RFs of a typical cell estimated just before and just after noise onset and offset. The preferred frequency of the cell was 6.7 KHz. The error bars represent 95% confidence bounds. The RFs were normalized to have the same peak value for plotting. The colors of the RFs correspond to the time intervals marked in a. c) The MTFs corresponding to the RFs in b. Before computing the MTFs, RFs were normalized such that the variance of the result of the convolution of the RF with the vocalization signal was one. d) The LF area of the MTFs for a sample of 6 cells, estimated at 200 ms intervals after noise onset and offset. The colored circles correspond to the time intervals marked in a. The results for each cell were normalized such that the LF area just before noise offset was 0 and the LF area just before noise onset was 1. The error bars represent one standard deviation of the distribution of normalized LF areas across the sample of cells.

To determine the time-course of the observed changes in temporal processing, we recorded responses while the stimulus was repeatedly switched between V and VN, and estimated temporal RFs at a range of times relative to noise onset or offset. A schematic illustration of the stimulus, which switched between V and VN every 3 seconds, is shown in figure 4a. Figure 4b shows the temporal RFs of a typical cell estimated just before and just after noise onset and offset. The RFs estimated just after noise onset (green) and 3 seconds after noise onset (red) are nearly identical, as are the RFs estimated just after noise offset (blue) and 3 seconds after noise offset (black). This is also evident in the MTFs shown in figure 4c, as the MTFs just after noise onset (green) and 3 seconds after noise onset (red) are lowpass, while the MTFs estimated just after noise offset (blue) and 3 seconds after noise offset (black) are bandpass. As shown in figure 4d, these results were consistent across a sample of 6 cells, as the LF area of the MTFs changed immediately (within 200 ms) following noise onset and offset and remained relatively constant until the next switch.

**Figure 5:**
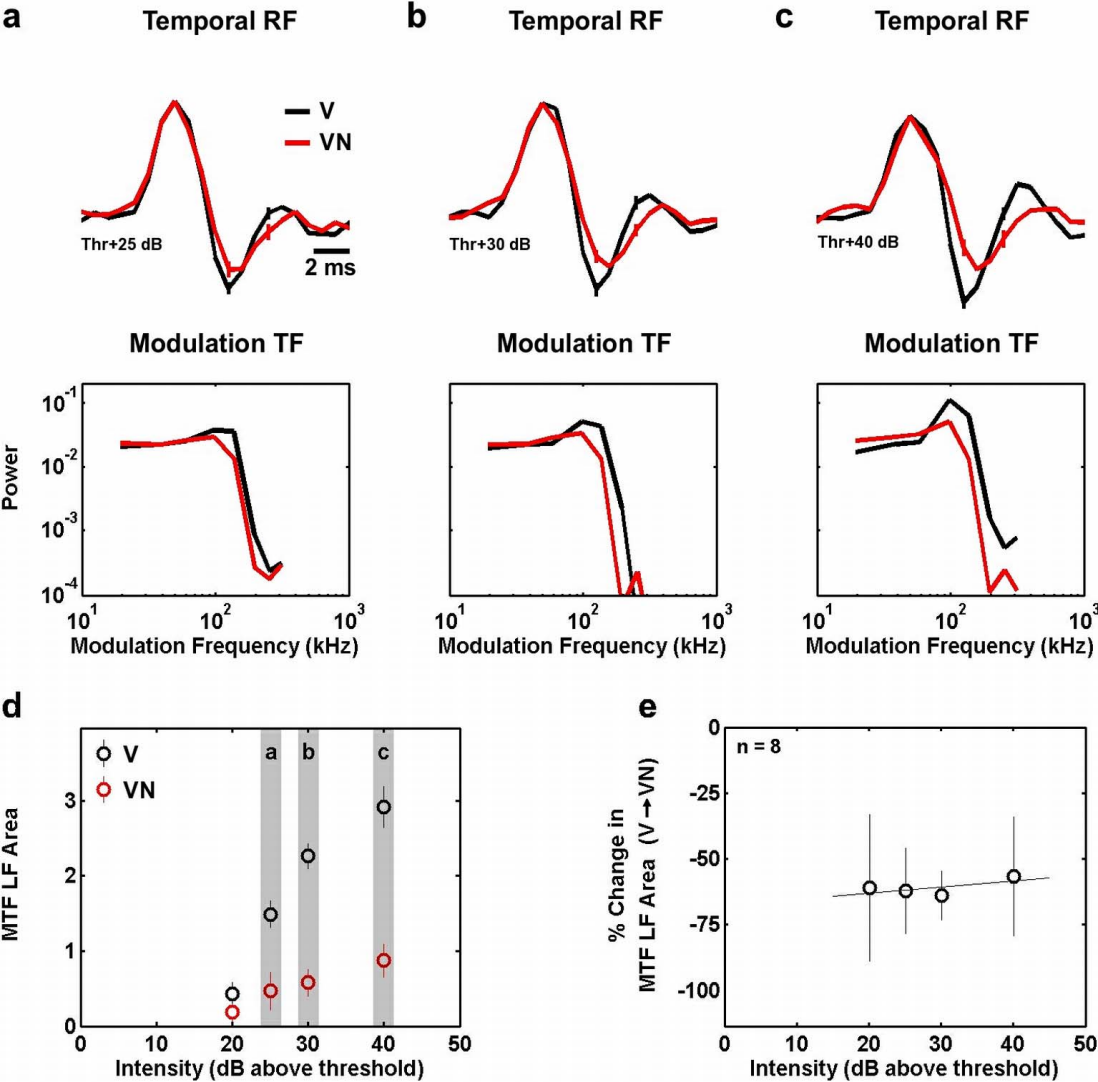
Context-dependent changes in temporal processing are robust to changes in the overall intensity of the stimulus. a–c) The RFs and MTFs for a typical cell estimated from responses to the V and VN stimuli at overall intensities of 25, 30, and 40 dB SPL above threshold. The preferred frequency of the cell was 10.1 KHz. The error bars represent 95% confidence bounds. The RFs were normalized to have the same peak value for plotting. Before computing the MTFs, RFs were normalized such that the variance of the result of the convolution of the RF with the vocalization signal was one. d) The LF areas of the V and VN MTFs at a range of intensities for the cell whose RFs and MTFs are shown in a–c. The error bars represent 95% confidence bounds. e) The percent change in LF area evoked by the addition of noise to the vocalization stimuli for a range of overall stimulus intensities for a sample of 8 cells. The error bars represent one standard deviation of the distribution of percent changes in LF area across the sample of cells.

We also examined the effects of overall stimulus intensity on the observed changes in temporal processing. We estimated temporal RFs from responses to the V and VN stimuli across a range of overall stimulus intensities, as shown for a typical cell at intensities of 25, 30, and 40 dB SPL above threshold in figures 5a–c. The increase in the overall intensity of the stimulus evokes an increase in the LF areas of both the V and VN MTFs, as shown in figure 5d. Accordingly, the magnitude of the decrease in the LF area of the MTF evoked by the addition of noise to the vocalization increased as the overall intensity of the stimulus increased. However, for a sample of 8 cells, the percent decrease in the MTF LF area evoked by the addition of noise remained relatively constant across a range of intensities, as shown in figure 5e. We also found that the observed changes in temporal processing were robust to changes in the spectral properties of the carrier of the vocalization stimulus, as shown in Figure S1.

### Changes in temporal processing promote efficient coding

**Figure 6:**
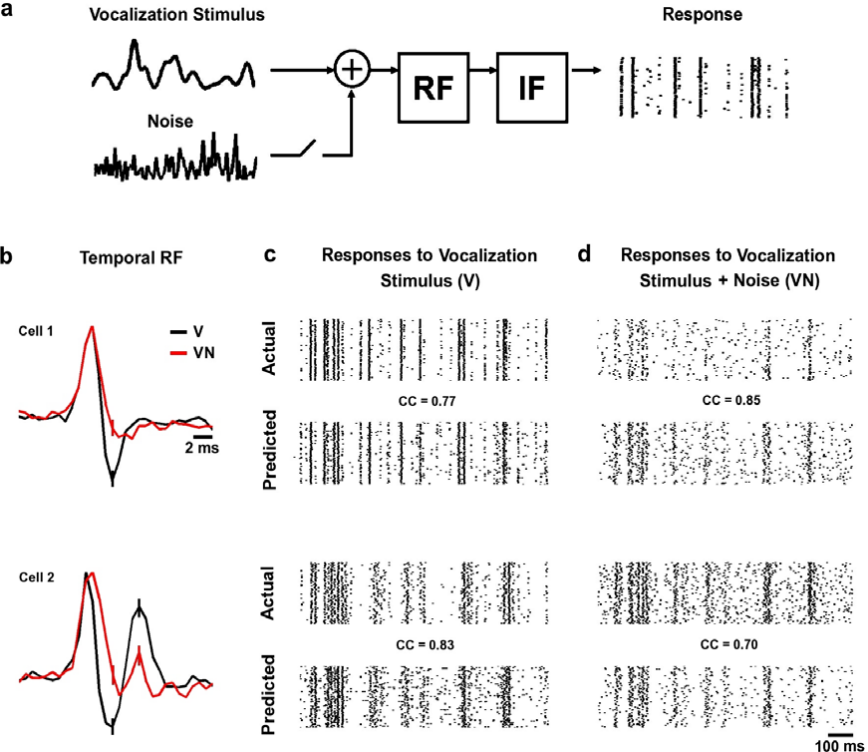
Actual and predicted responses of auditory neurons to naturalistic stimuli. a) A model of temporal processing in the auditory pathway. The vocalization signal (with or without additive noise) is passed through either the V or VN RF, and the result is fed into an integrate and fire mechanism to generate spikes. b) The temporal RFs estimated from responses to the V (black) and VN (red) stimuli for two cells with preferred frequencies of 12.5 and 3 KHz, respectively. The RFs were normalized to have the same peak value for plotting. c,d) The actual and predicted responses to repeated presentations of the V and VN stimuli for the two cells for which RFs are shown in b. The correlation coefficients between the predicted and actual responses are shown. The correlation coefficients were calculated for responses to novel stimuli (those not used to fit the model) for firing rate in 2 ms bins averaged over 100 repetitions.

The principles of efficient coding suggest that adaptive mechanisms should modulate a tradeoff between smoothing (increasing the SNR in the response) and whitening (reducing redundancy), with the optimal balance dependent on the overall SNR of the stimulus [7]. Viewed in this context, the results shown in figure 3 appear to capture the system at the two opposite sides of this tradeoff. Bandpass filtering in the V RF reduces the redundancy of the correlated (1/f) AMs in the vocalization stimulus by whitening at low frequencies, while lowpass filtering in the VN RF increases SNR under noisy conditions by preserving power at low modulation frequencies where the power in the vocalization stimulus is largest. To analyze the relative effectiveness of these two strategies, it is necessary to compare the responses to the vocalization stimulus with and without ambient noise when the system is using each processing strategy. Because of the fast time-course of the changes in temporal processing that we observe, as shown in figure 4, such a comparison cannot be made experimentally. Instead, we use a simple model in which the AMs in the stimulus are passed through either the V or VN RF for a particular cell, and the output of the RF is used to drive a leaky, noisy integrate and fire (IF) spike generator as shown in figure 6a. Figure 6b shows the V and VN RFs for two typical cells, and figures 6c and d show the actual responses of the cells to repeated presentations of the V and VN stimuli, along with the corresponding predicted responses of the model (with the temporal RF matched to the stimulus condition). For the subset of cells for which the model could be cross-validated on responses to novel stimuli (n = 23), the predictions were highly accurate, with correlation coefficients between predicted and actual responses of 0.62±0.09 for the V stimulus and 0.66±0.07 for the VN stimulus (for firing rate in 2 ms bins).

We first compared the responses of the model with the V and VN RFs to the vocalization stimulus alone. To ensure that the differences in the responses of the model with the V and VN RFs were the result of differences in the dynamics of the temporal RFs, the RFs were normalized such that their outputs had the same variance for a given stimulus condition. The spike trains produced by the model in response to repeated stimuli can be separated into signal (average response over all trials) and noise (deviation of individual trial from average response) components, as shown in 7a for a typical cell. With the only noise in the system being that of the IF mechanism, the power in the signal (thick lines) and noise (thin lines) in the responses are approximately equal at low frequencies. As expected from the relative shapes of the power spectrum of the AMs in the vocalization stimulus and the V and VN MTFs (the power spectrum of the signal in the response is related to the product of the stimulus power spectrum and the MTF), the response of the model with the V RF has reduced redundancy (the signal power is relatively flat at low frequencies), and the response of the model with the VN RF has an increased SNR at low frequencies.

**Figure 7:**
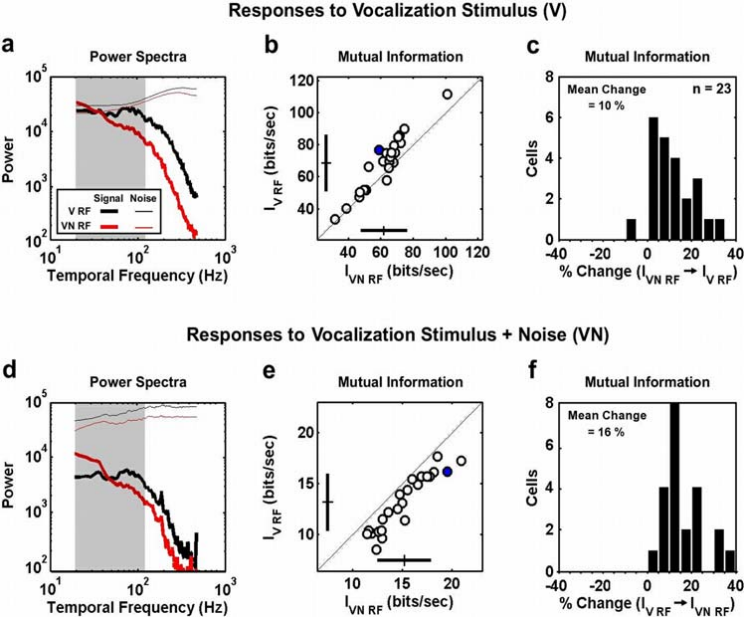
Context-dependent changes in temporal processing promote efficient coding. a) The power spectrum of the signal (thick lines) and noise (thin lines) in the responses of the model with the V (black) and VN (red) RFs to the vocalization stimulus alone for a typical cell (the same cell for which RFs and MTFs are shown in figures 3a and b). The gray band denotes the 20–120 Hz frequency range. The calculation of the signal and noise components of the responses is described in the Materials and Methods. b) The mutual information rate between the stimulus and the responses of the model with the V and VN RFs. The blue circle corresponds to the cell for which response spectra are shown in b. The crosses denote the sample mean±one standard deviation. c) A histogram showing the percent changes between the information rates shown in b (change from the responses of the VN model to the responses of the V model). d,e) Power spectra and information rates presented as in a and b for responses to the vocalization stimulus with ambient noise. f) A histogram showing the percent changes between the information rates shown in e (change from the responses of the V model to the responses of the VN model).

To quantify the efficiency of these responses, we computed the mutual information between the stimulus and response using the direct method, as shown in figure 7b. Across the sample of cells, the information rates resulting from processing in the V RF are significantly higher than those resulting from processing in the VN RF (paired t-test, p<0.01, n = 23), with increases as large as 33.2% and an average increase of 10.4±9.4% (see histogram in figure 7c). This result indicates that, in the absence of ambient noise, redundancy reduction through bandpass filtering in the V RF is, indeed, the preferred strategy. When the vocalization stimulus is combined with ambient noise, the shapes of the signal and noise power resulting from processing in the V and VN RFs remain the same, but the overall SNR in the response decreases, as shown in figure 7d. Because of this decrease in SNR, the information rates resulting from processing in the VN RF are now significantly higher than those resulting from processing in the V RF (paired t-test, p<0.01, n = 23, see figure 7e), with increases as large as 39.6% and an average increase of 16.4±10.5% (see histogram in figure 7f). This result suggests that, under noisy conditions, increasing SNR through lowpass filtering in the VN RF is the preferred strategy. Thus, in processing vocalization stimuli with and without noise, the responses of the model have a higher information rate when the RF in the model is matched to the stimulus condition, suggesting that the context-dependent changes in temporal processing that we observe may serve to promote efficient coding by increasing the information in the neural response.

## Discussion

We have shown that the temporal processing strategy employed by neurons in the auditory pathway is dependent on stimulus context. In response to the addition of noise to vocalization stimuli, we observed a change in the dynamics of the temporal RF that corresponds to a change from bandpass to lowpass tuning for AMs. These context-dependent changes in temporal processing occurred within a few hundred milliseconds of noise onset or offset, and were evident across a range of overall stimulus intensities. Using a simple model, we illustrated how these context-dependent changes in temporal processing enhance neural responses in a manner consistent with the principles of efficient coding by exploiting differences in the low-order statistical properties of vocalizations and sustained ambient noises.

It should be noted that we refer to the phenomena observed here as ‘adaptation’ not because of the mechanisms underlying it (which are not yet known), but because of its functional consequences: the temporal processing strategy (as represented by the temporal RF or MTF) changes in response to a change in the context of the stimulus. This is consistent with previous studies of adaptive processing in both the auditory and visual systems which report similar changes in temporal RFs in response to changes in the statistics of the relevant stimulus itself, for example, changes in the mean or variance of the stimulus [19], [20]. Unlike other forms of adaptation with observable temporal dynamics [21], [22], the phenomena observed here and in the studies cited above were evident almost immediately following a change in the statistics of the stimulus, making it impossible to determine (with RF based analyses) whether they reflect a truly adaptive mechanism with an extremely fast time-course or different modes of operation of a static (non-adaptive) nonlinear system. Regardless of whether or not the phenomena observed here are adaptive in a mechanistic sense, our results provide compelling evidence that the system is at least adaptive in a functional sense, as the changes in temporal processing evoked by a change in stimulus context appear to promote efficient coding.

### Relation to previous studies of adaptive temporal processing

Our results demonstrate that the addition of ambient noise to vocalization stimuli can evoke a change in the dynamics of the temporal RF that corresponds to a change from bandpass to lowpass tuning for modulation frequency. Similar phenomena were observed by Rees and colleagues as a result of the addition of a broadband noise masker to an amplitude modulated pure tone stimulus in the IC of the guinea pig and rat [13], [23]. However, our results differ from those of Rees and colleagues in several important ways. We report changes in modulation tuning in response to changes in the context of natural stimuli, rather than the addition of broadband white-noise to a white-noise modulated pure tone. In the study of adaptive temporal processing, the use of natural stimuli is essential in order to understand the functional consequences of the observed phenomena. Specifically, our results suggest that because of the differences in the modulation spectra of the ‘signal’ and ‘noise’ in our stimuli (vocalizations and ambient environmental noises), the changes in modulation tuning that we observe increase the information in the neural response. This increase in information would not be evident in the results of Rees and colleagues, because the modulation spectra of the ‘signal’ and ‘noise’ in their study are identical. The changes in modulation tuning observed by Rees and colleagues as a result of an increase in noise level cannot increase the information in the neural response (because the signal to noise ratio is the same at all modulation frequencies, there is no benefit in tuning in to any particular frequencies). The fact that Rees and colleagues observe changes in modulation tuning under stimulus conditions where the changes have no clear functional benefit has a very interesting implication, namely that the changes in modulation tuning are not based on any real-time measure of signal to noise ratio at different frequencies (in which case, Rees and colleagues would have observed no changes), but rather that the system has evolved based on the stereotypical properties of ‘signal’ and ‘noise’ in the natural environment.

In addition to describing the functional benefit of the observed phenomena within the context of natural stimuli, our results contain several additional novel observations. First, we observe that context-dependent changes in temporal processing are evident within 200 ms of a change in stimulus context (see Figure 4). This extremely fast time course implies that system is well suited to deal with rapid changes in stimulus context that may occur in the natural environment. Second, we demonstrate that the context-dependent changes in temporal processing are evident across a wide range of overall stimulus intensities (see Figure 5), again suggesting that the system is well suited to deal with simultaneous changes in stimulus context and intensity that may occur under natural conditions.

A recent study reported similar changes in temporal processing in the auditory system of awake songbirds in response to a change in the statistics of the relevant stimulus itself [24]. The study by Nagel and Doupe showed that a decrease in the overall intensity of a stimulus restricted to the neuron's preferred carrier frequency range causes a change from bandpass to lowpass modulation tuning, and we observed similar results in response to changes in the overall intensity of our stimuli (see figure 5). Nagel and Doupe observed changes in temporal processing within 100 ms of a change in stimulus intensity and suggested that this fast time-course reflects the presence of an adaptive nonlinearity. We observed a similar time-course for the context-dependent changes reported here (<200 ms, see figure 4), suggesting that a similar mechanism may underlie both phenomena. However, while adding noise to the vocalization stimulus increases its overall intensity, the changes in temporal processing that we observe (bandpass to lowpass) are actually in the *opposite* direction of those expected for an increase in intensity (lowpass to bandpass). This suggests that the changes we observe are due to stimulation at carrier frequencies outside the neuron's preferred range. Interestingly, the context-dependent effects that we observe were relatively consistent across a range of overall stimulus intensities (see figure 5) suggesting that adaptation to stimulus intensity and context may be functionally independent, as was recently observed for adaptation to mean luminance and contrast in the early visual pathway [22].

There have been a number of other studies of adaptive changes in temporal processing in the early visual system that reveal phenomena that are strikingly similar to those described above. Studies in the retina and lateral geniculate nucleus have shown that a decrease in the mean luminance or contrast of the visual stimulus causes a change in the dynamics of the temporal RF, corresponding to a change from bandpass to lowpass tuning for temporal frequency [25]–[30]. The time-course of these changes is similar to that observed for the corresponding changes in the auditory system, occurring within 100 ms of a change in mean luminance or contrast [31] and there is evidence that these changes may serve to maintain the flow of visual information [32]. These similarities in adaptive temporal processing across sensory modalities suggest that efficient coding may be a general strategy employed by sensory systems.

### Temporal processing and efficient coding

It is interesting to note that both the context-dependent changes in temporal processing that we observed here and the adaptive changes that have been observed in previous studies in response to changes in the intensity of the relevant stimulus itself are predicted by the efficient coding hypothesis [7]. Because the power spectra of behaviorally relevant stimuli typically decrease with increasing modulation (or temporal) frequency while the power spectra of background noises are relatively flat (see figure 1 and Singh and Theunissen [33]), the SNR of the combined behaviorally relevant and background noise stimuli decreases with increasing temporal frequency. The efficient coding hypothesis states that the optimal strategy for temporal processing depends on the overall SNR of the stimulus, with modulation tuning changing from bandpass to lowpass as the overall SNR is decreased. When the overall SNR is high, bandpass filtering can increase information through whitening (decreasing the redundancy of correlated stimuli). When the overall SNR is low, lowpass filtering can increase information through smoothing (conserving signal power at low frequencies where the SNR is high). Since both a decrease in overall intensity of the stimulus and the addition of noise correspond to a decrease in the overall SNR of the stimulus, the efficient coding hypothesis predicts that both of these changes will evoke a transition from bandpass to lowpass modulation tuning, in agreement with the experimental results. Thus, intensity- and context-dependent changes in temporal processing can be unified under the efficient coding hypothesis.

### Possible mechanisms underlying changes in temporal processing

Recent studies have revealed a number of the mechanisms that underlie adaptive temporal processing in the early visual pathway [34]–[36], but the mechanisms that underlie adaptive temporal processing in the auditory system are not yet known. Our results demonstrate that the addition of ambient noise to vocalization stimuli causes a change in the dynamics of the temporal RF that corresponds to a change from bandpass to lowpass tuning for modulation frequency. Given the existing evidence that inhibition shapes temporal processing in the auditory midbrain and brainstem, it is likely that inhibition also modulates the context-dependent changes that we observe here [37]–[39]. Indeed, a previous study of the effects of inhibition on modulation tuning in the chinchilla IC showed that blocking GABA *_A_* could cause a change from bandpass to lowpass modulation tuning [40]. However, given the abundance of inhibitory connections in the auditory periphery, we can only speculate as to whether the changes in temporal processing that we observe reflect changes in brainstem circuitry or are generated *de novo* in the IC. Further study is necessary to determine the locus of context-dependent changes in temporal processing in the auditory pathway and to define the precise role of inhibition in this context.

## Materials and Methods

### Analysis of natural sounds

Animal vocalizations and bird songs from 30 different species (for example: falcon, wren, chickadee, porcupine, lemur, mink) were provided by the Library of Natural Sounds, Cornell Library of Ornithology, Cornell University. Up to 3 seconds of sounds from each species were concatenated to produce 80 seconds of continuous sound. Human speech was taken from the IViE Corpus, Department of Linguistics, University of Cambridge. Read passages from several English speakers were concatenated to produce 80 seconds of continuous sound. The animal vocalizations, bird songs, and speech were then concatenated to produce a 160 second ensemble of vocalizations. Ambient noise sounds were taken from the Freesound Project, Universitat Pompeu Fabra. Sounds from a variety of sources (rain, wind, waterfall, large crowd, vacuum cleaner) were concatenated to produce a 160 second ensemble of ambient noises. Example sounds from each ensemble are shown in Figure S1. The sampling rate for all sounds was at least 44.1 KHz.

To analyze the statistics of the amplitude modulations (AMs) in vocalization and ambient noise sound ensembles, all sounds were converted to spectrograms with a carrier frequency resolution of 460 Hz and a temporal resolution of 1 ms. AMs were extracted from each frequency band between 2.5 and 17.5 KHz (this range included the preferred frequencies for >90% of the cells in this study) for each sound and normalized to have a minimum value of 0 and a maximum value of 1. These normalized AMs were used to compute amplitude distributions and power spectra as shown in figures 1c and d. The amplitude distribution of AMs in vocalizations was well described by an exponential distribution with *λ* = 0.015 (averaged across all carrier frequencies) and the amplitude distribution of AMs in ambient noise was well described by a Rayleigh distribution with *β* = 0.1 (averaged across all carrier frequencies). The power spectra of the AMs were fit the function 1/*f^α^* in the 20–120 Hz frequency range, and the best fits were achieved with *α* = 1.4 for vocalizations and 0.1 for ambient noise (averaged across all carrier frequencies). These values for the parameters of the amplitude distributions and power spectra of the AMs were used to create the modulation signals for experimental stimuli as described below. These results are qualitatively similar to those of a previous study of the statistical properties of AMs in different classes of natural sounds [41]. The quantitative differences between our results and those of Singh and Theunissen are likely due to the specifics of the sound ensembles used (for example, Singh and Theunissen included both transient and sustained environmental noises).

### Creation of naturalistic stimuli

We used the amplitude distributions and power spectra of the AMs in vocalizations and ambient noise described above to create modulation signals for naturalistic stimuli. The processes for the creation of the vocalization and ambient noise signals were slightly different. First, to create the vocalization signal, Gaussian white noise was filtered to have a power spectrum of 1/*f^α^* with *α* = 1.3 (*α* was increased to the desired value of 1.4 by the logarithmic operation described below). Next, the logarithm of the signal was taken (after adding a constant value to the signal so that its minimum value was 1) and the result was scaled such that its distribution was exponential with *λ* = 0.015. To create the ambient noise signal, two Gaussian white noise signals were filtered to have power spectra of 1/*f^α^* with *α* = 0.1. Next, each signal was squared, and the square root of the sum of these squared signals was taken and raised to the power of 0.1, such that the distribution of the resulting signal was Rayleigh with *β* = 0.1. For the vocalization stimulus (V), the vocalization signal was used to modulate a pure tone carrier at a neuron's preferred frequency (and also, for a subset set of cells, a broadband pink noise carrier). For the vocalization stimulus with ambient noise (VN), the vocalization stimulus was combined with broadband Gaussian noise modulated by the ambient noise signal. The SNR in the VN stimulus was −10 dB.

### Physiological recordings

The surgical procedures used in this study have been described in detail previously [42]. All experiments were approved according to the German Tierschutzgesetz (AZ 211-2531-40/01+AZ 211-2531-68/03). Briefly, adult Mongolian gerbils (Meriones unguiculatus) were anesthetized by an initial intraperitoneal injection (0.5 ml/100 g body weight) of a physiological NaCl solution containing ketamine (20%) and xylazine (2%). During surgery and recordings, a dose of 0.03 ml of the same mixture was applied subcutaneously every 20 min. A small metal rod was mounted on the frontal part of the skull and used to secure the head of the animal in a stereotactic device during recordings. The animal was positioned in a sound-attenuated chamber and a craniotomy was made over the inferior colliculus, 1.3–2.6 mm lateral from the midline and 0.5–0.8 mm caudal from the bregma. The dura mater overlying the cortex was removed, and glass electrodes filled with 1 M NaCl (5–15 *M*Ω) were advanced into the inferior colliculus (2–4 mm below the surface).

Extracellular action potentials were recorded, filtered, and fed into a computer via an A/D converter (RP2-1, TDT). Clear isolation of action potentials from single-units was achieved by off-line spike cluster analysis (Brainware, Jan Schnupp, TDT). Typical recording periods lasted 10–14 h. After recordings, the animal was killed without awakening by an injection of 0.1 ml of barbital. For some animals, the last electrode position was marked by a pressure-induced injection of Dextran and recording sites were verified to be in the central nucleus of the inferior colliculus using standard histological techniques [43].

### Acoustic stimulation

Stimuli were generated at a 50 KHz sampling rate by TDT System III (Tucker Davis Technologies). Digitally generated stimuli were converted to analog signals (RP2-1, TDT), attenuated (PA5, TDT) and delivered to electrostatic speakers (EC1, TDT) coupled to tubes which were inserted in the ears. All stimuli were presented monaurally to the ear contralateral to the recording site. When searching for cells, repeated presentations of a 200 ms segment of broadband noise were presented. When a single-unit was isolated, 200 ms pure tones of various intensities and frequencies were presented to determine the preferred frequency and threshold. Only those neurons with sustained responses to the pure tone stimulus (those that responded on average with more than one spike in the last 150 ms of a 200 ms pure tone at the preferred frequency, 20 dB above threshold) were tested with the V and VN stimuli described above (n = 78). For all neurons, non-repeating 40 second segments (1–10 repetitions) of the V and VN stimuli were presented and responses were used for estimation of temporal RFs. Only those neurons whose firing rate was strongly modulated by the V and VN stimuli were included in the study (n = 63). Strong modulation was defined by the peak of temporal RF being at least 10 times larger than the noise level as determined by one standard deviation of the temporal RF estimated from shuffled responses. The preferred frequencies and thresholds for these neurons ranged from 2.5–24 KHz and 25–65 dB SPL. For most neurons (n = 40), we also recorded responses to repeated tone bursts at the preferred frequency (25 dB above threshold) to characterize response type. Of these neurons, 57% were choppers, 20% were onset-sustained, 15% were pausers, and 8% were sustained, according to the criteria outline by Rees et al. (REES97). For a subset of neurons (n = 23), responses to repeated presentations of short segments (3 sec, 100 repetitions) of the V and VN stimuli were also recorded and used for fitting and testing the model of auditory temporal processing as described below. Each repetition of the VN stimulus used the same vocalization signal and a different noise signal. The distributions of response properties (best frequency, threshold, mean firing rate, SNR) across this subset of neurons were not significantly different from those across the rest of the population (t-tests, p>0.1). The V stimulus was presented with peak intensity between 45–85 dB SPL (the mean intensity was approximately 25 dB lower) depending on the threshold of the neuron. The additional noise in VN stimulus with the SNR described above raised the peak intensity by 3 dB SPL (and the mean intensity by 10 dB SPL).

### Estimation of receptive fields and modulation tuning functions

Temporal RFs were estimated via least-squares estimation. To characterize temporal processing, the vocalization modulation signal itself was used for RF estimation, rather than the full spectrotemporal V or VN stimulus. The vocalization signal alone was used for estimation of both V and VN RFs, as the resulting estimates of the VN RF were less noisy than those estimated from the combined vocalization and noise signals, with no observable bias. We denote the (zero mean) modulation signal as *s*[*n*], where *n* is the time sample. We denote the RF as *g*[*m*], representing a non-parametric temporal RF with *M* lags. We assume a linear relationship between stimulus and response such that the response at time *n* is given by the convolution of the *M* preceding stimuli with the RF. This convolution can be written as a matrix multiplication: *r*[*n*] = *s_n_ g*, where
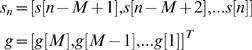
and *T* denotes matrix transpose. Similarly, the stimulus/response relationship for a record of *N* time steps can be written as *R* = *S g*, where
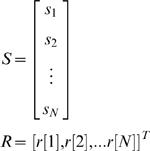



As described by Theunissen and colleagues [44], the RF 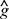 that minimizes the mean squared error between the actual response *R* and the estimated response 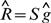 is given by:
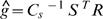
where *C_s_* is the autocorrelation matrix of the stimulus:
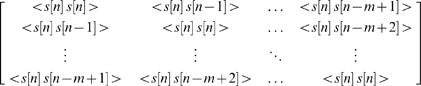
where <.> denotes the average across all stimuli. In this case, the least-squares estimate of the RF 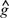 is equal to the so-called ’reverse-correlation’ estimate of the RF, *S ^T^ R*, multiplied by the inverse of the stimulus autocorrelation matrix *C_s_* ^−1^. The correlations in the stimulus can bias the reverse-correlation estimate of the RF and multiplication by *C_s_* ^−1^ corrects this bias. However, it should be noted that the least-squares technique described here only corrects for the bias introduced by the second-order statistics of the stimulus. To ensure that the higher-order statistical properties of the stimuli used in this study did not introduce an additional bias into the RF estimate, we simulated responses to the V and VN stimuli with a known RF (using the model described below). The least-squares estimate of the RF from these simulated responses was an unbiased version of the original RF, indicating that the higher-order statistical properties of the stimuli did not bias the estimate.

Because of the correlations in naturalistic stimuli, the singular values of *C_s_* can decrease rapidly (the condition number may be high), and its inversion may be ill-conditioned. However, as we have described previously [45], [46], the above equation can be solved recursively, starting with an initial value for *C_s_* ^−1^ and 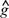 and improving the estimates with each new stimulus/response observation. At each time step, the RF estimate computed from previous data 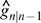 is used to generate a linear prediction of the response of the neuron to the new stimulus (the subscript *n*|*n*−1 denotes an estimate at time *n* given all data up to and including time *n*−1). This prediction is compared with the actual response *r*[*n*] to yield the prediction error 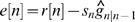. The RF estimate is updated by scaling the error by a gain factor related to the stimulus autocorrelation matrix 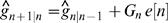. The gain is computed at each time step as follows:
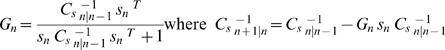



Data from all times within the stimulus/response record are weighted equally in the estimation of 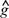.

The RF estimate 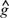 can be further improved through regularization [47]. To initialize the algorithm, the initial conditions 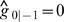 and 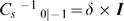 are used. The regularization parameter *δ* effectively reduces the condition number of *C_s_* by adding a constant to all of the elements along the diagonal of *C_s_* ^−1^. However, this manipulation also introduces a bias into the RF estimate, and the value of *δ* must be chosen to optimize the tradeoff between error avoided by decreasing the condition number of *C_s_* and error introduced by biasing the RF estimate. For this study, we estimated RFs with a range of values for *δ* and chose the value (*δ* = 0.001) which produced the RFs with the most predictive power for the neurons for which RFs could be cross-validated with responses to novel stimuli. For RF estimation, spike times were binned to give an estimate of the firing rate in 1 ms bins. RFs were also estimated from shuffled responses, and the standard deviation of these estimates were used to set confidence bounds. Modulation tuning functions (MTFs) were obtained by computing the power spectrum of the estimated temporal RFs.

### A model of temporal processing in the auditory pathway

To simulate responses to the V stimulus, the vocalization modulation signal is passed through either the V or VN RF, estimated as described above, and the output is used to drive and a leaky, noisy integrate and fire (IF) spike generator. To simulate responses to the VN stimulus, the vocalization and ambient noise modulation signals were combined before passing through the RF. This model is based on the one used by Escabi et al. [48] to predict the responses of neurons in the cat IC to complex auditory stimuli.

The potential of the IF mechanism was governed by the following differential equation:
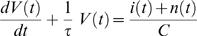
where V(t) is the difference between the membrane potential and its resting potential, i(t) is the output of the temporal RF, n(t) is Gaussian white noise (representing the internal noise evident in variable responses to identical stimuli), *τ* is the membrane time constant, and C is the membrane capacitance. Spikes are generated in the model whenever the membrane potential exceeds a specified threshold. After activation, a 2 ms refractory period was imposed and the membrane was reset to its resting potential. Because the temporal RFs include the effects of temporal integration in the cell membrane, the output of the RF was inverse filtered with the membrane impulse response of the cell, *h*(*t*) = *C* ^−1^ e^−t/*τ*^, before being fed into the IF mechanism. To simplify the fitting of the model to experimental data, we fixed the membrane time constant at 10 ms, and defined a dimensionless ‘normalized threshold’ that defined the spike threshold in terms of the standard deviation of the intracellular potential (relative to the resting potential). Thus, the response of the neuron was independent of the scaling of the input and it was only necessary to fit two parameters, the normalized threshold and the ratio of the internal noise *n*(*t*) to the output of the RF *i*(*t*). These parameters were fit by maximizing the correlation coefficient between the simulated response of the model and the actual response of each neuron (for firing rate in 2 ms bins), using temporal RFs that were matched to the stimulus condition. For the sample of cells studied here, the optimal values of threshold and SNR parameters ranged from 0.6 to 1.8 and −6 to 10 dB.

The model was used to simulate the response of the system with different temporal processing strategies using the V and VN RFs. To isolate the effects of changes in temporal processing, the V and VN RFs were normalized such that their outputs had the same variance for a given stimulus condition, and the parameters of the IF mechanism were matched to the stimulus condition, independent of which RF was used.

### Calculation of response power spectra, signal to noise ratio, and mutual information

For a subset of cells, we recorded responses to short repeated segments of the V and VN stimuli as described above. The SNRs of these responses were calculated (for firing rate in 1 ms bins) as described by Borst and Theunissen [49]. First, the signal spectrum is obtained by computing the power spectrum of the response after averaging across all trials. Next, to obtain the noise power, the response from each trial is subtracted from the average response and the power spectrum of this difference is computed. These difference spectra are averaged over all trials to yield the overall noise spectrum. Finally, the SNR is given by the ratio of the total power of the signal and noise spectra.

The same procedure was used to calculate the signal and noise spectra of simulated responses to 512 repeats of 30 second segments of the V and VN stimuli. These simulated responses were also used to calculate the mutual information between the stimulus and response using the direct method [50]. Each spike train was binned with *δt* = 1 ms resolution. The total entropy (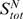, in bits/sec) of the probability distribution of possible N-bit words (*P*(*w*)) was measured as:
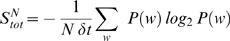



The noise entropy (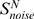, in bits/sec) of the conditional probability distribution of possible N-bit words (*P*(*w*|*t*)) at a given time in the experiment was measured as:
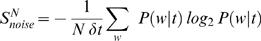
and averaged over all values of *t*. Total and noise entropies were measured for N = [3], [4], [5], [6] and extrapolated to N = ∞. The difference between these extrapolated values 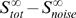 gives the mutual information in the response. Calculations with different stimulus lengths and numbers of repeats were used to verify the stability of the information measures.

## Supporting Information

Figure S1 Context-dependent changes in temporal processing are robust to changes in the spectral properties of the carrier of the vocalization stimulus. To test whether our observations of context-dependent temporal processing were dependent on the spectral properties of the carrier of the vocalization stimulus, we estimated temporal RFs from responses to the V and VN stimuli with both a pure tone and a pink noise carrier for the vocalization stimulus. The power spectrum of the pink noise carrier was 1/f̂a with a = −1 in the 2.5–24 KHz frequency range, and the total power of the pure tone and pink noise carriers were equal. Results for a typical cell are shown in panel a. For both the pure tone and pink noise carriers, the addition of noise causes a clear change in temporal processing. Panel b shows the average LF areas of the V and VN MTFs with pure tone and pink noise carriers for a sample of 9 cells (error bars represent one standard deviation). The LF areas were typically larger for the pure tone carrier than for the pink noise carrier during both V and VN stimulation. While the pure tone and pink noise stimuli had the same overall power, the broadband nature of the pink noise carrier results in much of its power being outside the range of frequencies to which an individual neuron is responsive, while the power in the pure tone carrier is always concentrated at the frequency to which the neuron is most responsive. This difference in effective power may be the reason the LF areas of the MTFs are larger for the pure tone carrier than for the pink noise carrier, as the LF area of the MTF is known to increase with increased stimulus power (see figure 5 and Nagel and Doupe, Neuron, 2006). Nonetheless, across the sample of cells, the addition of noise still results in a large decrease (29%) in the LF area of the MTFs as shown in panel c, indicating that the effects of ambient noise on temporal processing are robust to changes in the spectral properties of the carrier of the vocalization stimulus.
(0.27 MB TIF)
