# Supplementary figures and images for: Efficient Temporal Processing of Naturalistic Sounds
Source: PLoS One. 2008 Feb 27;3(2):e1655. doi: 10.1371/journal.pone.0001655 (PMC2249929; doi:10.1371/journal.pone.0001655)

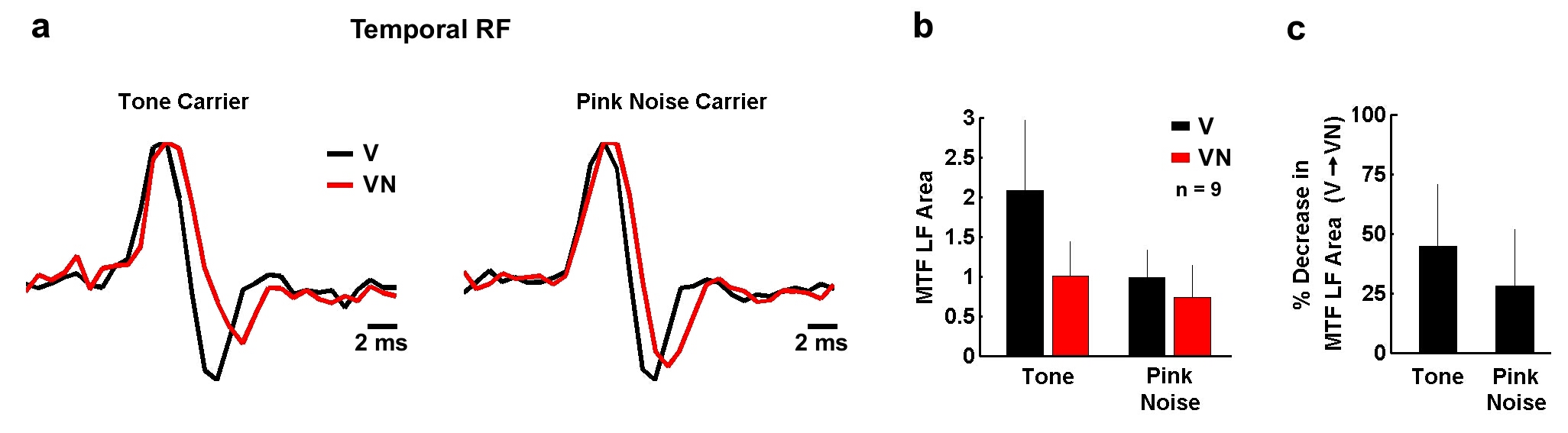

Supplement: Figure S1 — Context-dependent changes in temporal processing are robust to changes in the spectral properties of the carrier of the vocalization stimulus. To test whether our observations of context-dependent temporal processing were dependent on the spectral properties of the carrier of the vocalization stimulus, we estimated temporal RFs from responses to the V and VN stimuli with both a pure tone and a pink noise carrier for the vocalization stimulus. The power spectrum of the pink noise carrier was 1/f̂a with a = −1 in the 2.5–24 KHz frequency range, and the total power of the pure tone and pink noise carriers were equal. Results for a typical cell are shown in panel a. For both the pure tone and pink noise carriers, the addition of noise causes a clear change in temporal processing. Panel b shows the average LF areas of the V and VN MTFs with pure tone and pink noise carriers for a sample of 9 cells (error bars represent one standard deviation). The LF areas were typically larger for the pure tone carrier than for the pink noise carrier during both V and VN stimulation. While the pure tone and pink noise stimuli had the same overall power, the broadband nature of the pink noise carrier results in much of its power being outside the range of frequencies to which an individual neuron is responsive, while the power in the pure tone carrier is always concentrated at the frequency to which the neuron is most responsive. This difference in effective power may be the reason the LF areas of the MTFs are larger for the pure tone carrier than for the pink noise carrier, as the LF area of the MTF is known to increase with increased stimulus power (see figure 5 and Nagel and Doupe, Neuron, 2006). Nonetheless, across the sample of cells, the addition of noise still results in a large decrease (29%) in the LF area of the MTFs as shown in panel c, indicating that the effects of ambient noise on temporal processing are robust to changes in the spectral properties of the carrier of the voc [file pone.0001655.s001.tif]
